# Astaxanthin Attenuates Chlorpyrifos-Induced Pulmonary Cytotoxicity by Modulating Mitochondrial Redox and Inflammatory Pathways

**DOI:** 10.3390/cimb47080663

**Published:** 2025-08-17

**Authors:** Mediha Demet Okudan Altındaş, Adem Güner

**Affiliations:** 1 Hamidiye Faculty of Health Sciences, Department of Occupational Health and Safety, University of Health Sciences, 34668 İstanbul, Türkiye; 2 Faculty of Health Sciences, Department of Occupational Health and Safety, Sinop University, 57000 Sinop, Türkiye

**Keywords:** astaxanthin, chlorpyrifos, lung, mitochondrial dysfunction, oxidative stress, toxicity

## Abstract

Chlorpyrifos (CPF), an organophosphate pesticide, is known to induce pulmonary toxicity through oxidative stress, mitochondrial dysfunction, and inflammation. Astaxanthin (ASX), a xanthophyll carotenoid derived primarily from marine microalgae (Haematococcus pluvialis), possesses strong antioxidant properties and has demonstrated cellular protective effects in numerous oxidative stress studies. However, its efficacy against CPF-induced lung cell damage remains uncharacterized. This study revealed the protective role of ASX, as a pretreatment and co-treatment, against CPF-induced cytotoxicity in human A549 lung adenocarcinoma cells by assessing cell viability, intracellular reactive oxygen species (IROS), total oxidative status (TOS), total antioxidant capacity (TAC), mitochondrial membrane potential (MMP), intracellular calcium ions (Ca^2+^), lactate dehydrogenase (LDH) release, malondialdehyde (MDA) levels, glutathione peroxidase (GPx) activity, superoxide dismutase (SOD) activity, DNA fragmentation, and apoptosis/inflammation-associated gene expression. CPF treatment significantly decreased cell viability and TAC, while elevating IROS, TOS, MMP, intracellular Ca^2+^, and LDH release. CPF also increased MDA levels and suppressed GPx and SOD activities. DNA fragmentation and quantitative polymerase chain reaction (qPCR) analysis revealed upregulation of pro-apoptotic and inflammatory markers such as BCL2-associated X protein (BAX), caspase-3 (CASP3), tumor protein p53 (TP53), tumor necrosis factor-alpha (TNF-α), interleukin-1 beta (IL-1β), nuclear factor kappa B (NFκB), and voltage-dependent anion-selective channel protein 1 (VDAC1) and suppression of anti-apoptotic B-cell lymphoma 2 (BCL2) and antioxidant defense genes nuclear factor erythroid 2-related factor 2 (Nrf2) and heme oxygenase-1 (HO-1). ASX treatment, particularly when administered as a pretreatment, significantly reversed CPF-induced oxidative and inflammatory responses by restoring SOD, GPx, and TAC levels, reducing IROS, TOS, MDA, and LDH release, and downregulating apoptotic and inflammatory gene expressions. ASX pretreatment notably decreased MMP and intracellular Ca^2+^ levels, indicating protection against mitochondrial dysfunction and calcium dysregulation. ASX upregulated Nrf2 and HO-1 expression and restored the BCL2/BAX balance, suggesting inhibition of mitochondrial-mediated apoptosis. Additionally, ASX significantly attenuated CPF-induced anti-angiogenic effects in the in ovo Hen’s Egg Test Chorioallantoic Membrane (HET-CAM) assay. These findings demonstrate, for the first time, that ASX exerts a broad spectrum of protective effects against CPF-induced cytotoxicity in lung cells, mainly through the stabilization of mitochondrial redox status and modulation of apoptosis- and inflammation-related gene pathways, highlighting ASX as a promising candidate for further therapeutic development. Furthermore, the pronounced efficacy observed in the pretreatment regimen suggests that ASX can be evaluated as a potential nutritional preventive strategy in high-risk populations with occupational or environmental CPF exposure.

## 1. Introduction

Organophosphate compounds are among the most widely used classes of pesticides in global agriculture, applied extensively to protect crops such as cotton, corn, rice, vegetables, and fruits. Chlorpyrifos (CPF) (O,O-diethyl O-3,5,6-trichloro-2-pyridyl phosphorothioate) is a widely used broad-spectrum organophosphate insecticide, introduced in the 1960s, that remains one of the most extensively applied and toxic compounds within this chemical class [1,2]. Despite regulatory restrictions in several countries, CPF continues to be utilized in many regions owing to its low cost, high pesticidal efficacy, and broad-spectrum activity. However, the environmental persistence, bioaccumulation potential, and long-term ecological and human health implications of CPF exposure have raised significant public health concerns [3].

CPF exhibits pronounced acute and chronic toxicity, predominantly affecting the nervous and respiratory systems. Human exposure primarily occurs via inhalation, ingestion, and dermal absorption, with occupational settings and environmental contamination constituting the principal sources [4]. Following systemic absorption, CPF is bioactivated in the liver to CPF-oxon, an irreversible acetylcholinesterase inhibitor that induces cholinergic overstimulation. In addition to AChE inhibition, CPF triggers oxidative stress, mitochondrial dysfunction, and inflammatory signaling in pulmonary tissues, contributing to airway hyperresponsiveness, epithelial injury, inflammation, and structural remodeling [5,6,7]. Individuals in agricultural practices, pesticide application, and greenhouse operations face elevated exposure risks, particularly via aerosolized particles and contaminated dust. Biomonitoring studies have confirmed systemic uptake of CPF through the detection of its primary urinary metabolite, 3,5,6-trichloro-2-pyridinol (TCPy), in exposed populations. Chronic occupational exposure correlates with reduced forced expiratory volume in one second (FEV_1_) and forced vital capacity (FVC), indicative of progressive obstructive lung disease [8,9]. Clinical reports indicate that approximately 25% of acutely exposed individuals require ventilatory support or succumb to poisoning-related complications [10]. Experimental in vivo studies have shown CPF-induced histopathological alterations in bronchiolar and alveolar structures, including epithelial desquamation, inflammatory cell infiltration, and interstitial edema, accompanied by elevated levels of malondialdehyde (MDA), tumor necrosis factor-alpha (TNF-α), and nuclear factor kappa B (NF-κB) activation [11,12]. At the molecular level, CPF disrupts redox homeostasis through excessive reactive oxygen species (ROS) generation, mediated by mitochondrial complex I inhibition and NADPH oxidase activation. This oxidative burden results in lipid peroxidation, glutathione depletion, and impairment of endogenous antioxidant systems [13,14]. CPF can also induce oxidative DNA damage and modulate redox-sensitive pathways involved in lung cancer cell survival, potentially contributing to carcinogenic progression [15]. Given the lack of specific antidotes beyond cholinesterase reactivators and supportive care, antioxidant-based interventions aimed at scavenging ROS and restoring redox balance represent a promising therapeutic approach [16].

Astaxanthin (ASX; 3,3′-dihydroxy-β,β-carotene-4,4′-dione) is a lipophilic xanthophyll carotenoid naturally synthesized by various microalgae species, including *Haematococcus pluvialis* and *Chlorella zofingiensis*, as well as by the yeast *Phaffia rhodozyma* and certain crustaceans. Its molecular structure, characterized by a conjugated double-bond system and polar terminal hydroxyl and keto groups, confers exceptional antioxidant capacity, enabling effective scavenging of ROS and protection of lipid membranes from oxidative damage [17]. ASX exhibits superior radical-scavenging activity compared to α-tocopherol and β-carotene by approximately 100- and 500-fold, respectively [18], attributed to its ability to span the lipid bilayer and function at both polar and nonpolar interfaces [19]. This amphipathic nature supports stabilization of cellular membranes and redox regulation. ASX is also the only ketocarotenoid reported to cross the blood–brain barrier via transcytosis, enhancing its systemic therapeutic potential [20]. Preclinical and clinical studies have demonstrated ASX’s anticancer, neuroprotective, cardioprotective, and immunomodulatory properties [19,20,21]. In pulmonary contexts, ASX attenuates oxidative stress and inflammation through modulation of Nrf2/HO-1, NF-κB, MAPK, JAK/STAT3, and PI3K/Akt pathways [22,23], and regulates immune cell activation and cytokine release. It mitigates alveolar injury in lipopolysaccharide-induced acute lung injury and suppresses tissue destruction in cigarette smoke–induced chronic obstructive pulmonary disease (COPD) and emphysema models [23,24]. ASX also protects respiratory epithelial cells from environmentally persistent free radical (EPFR)-induced damage by reducing mitochondrial ROS production, stabilizing mitochondrial function, restoring antioxidant signaling, and modulating genes involved in mucin production (e.g., MUC5AC), apoptosis, inflammation, and mitophagy [25]. In cadmium-induced toxicity models, ASX alleviated ROS-mediated cytotoxicity and inhibited pro-migratory signaling in lung epithelial cells [26]. Evidence from aquatic models supports its systemic antioxidant efficacy, as dietary ASX improved growth performance and reduced paraquat-induced oxidative damage in rainbow trout by inhibiting protein and lipid peroxidation while upregulating antioxidant-related genes [27]. Collectively, these findings underscore ASX’s therapeutic potential as a bioactive compound capable of modulating oxidative and inflammatory pathways, particularly in respiratory diseases characterized by redox imbalance.

To the best of our knowledge, this is the first study to investigate the protective role of ASX against CPF-induced cytotoxicity in human lung epithelial cells by comprehensively examining the mitochondrial–calcium–redox axis and directly comparing pretreatment and co-treatment strategies. The adenocarcinomic human alveolar basal epithelial cells (A549) were selected because they closely mimic the cellular environment of the respiratory tract, which is the primary route of CPF exposure in both occupational and environmental settings. Therefore, it represents as a relevant in vitro model for assessing CPF-induced pulmonary toxicity. To this end, this study aimed to reveal the protective effect of ASX against CPF-induced toxicity in A549 by evaluating cell viability (3-(4,5-dimethylthiazol-2-yl)-2,5-diphenyltetrazolium bromide assay [MTT] and lactate dehydrogenase release [LDH]), oxidative and antioxidant status (superoxide dismutase [SOD], glutathione peroxidase [GPx], malondialdehyde [MDA], total oxidant status [TOS], and total antioxidant capacity [TAC], intracellular ROS [IROS]), mitochondrial membrane potential (MMP), intracellular calcium (Ca^2+^) levels, DNA fragmentation, apoptosis/inflammation-related gene expression, and in ovo Hen’s Egg Test Chorioallantoic Membrane (HET CAM) anti-angiogenic effects.

## 2. Materials and Methods

### 2.1. Cell Culture, Viability, and DAPI Staining

The human alveolar basal epithelial A549 cell line (ATCC CCL-185) was obtained from the American Type Culture Collection (ATCC, Manassas, VA, USA). Cells were cultured in DMEM supplemented with 10% fetal bovine serum (FBS) and 1% penicillin-streptomycin and incubated at 37 °C in a humidified atmosphere containing 5% CO_2_.

For the experiments, A549 cells were seeded into 96-well microplates at a density of 1 × 10^5^ cells/mL and allowed to adhere for 24 h. Cells were then subjected to the following treatment groups: Control group, cells treated with 400 μM CPF alone for 24 h; cells treated with 20 μM ASX alone for 24 h; cells pretreated with 20 μM ASX for 6 h, followed by 400 μM CPF exposure for an additional 24 h; and cells co-treated with 20 μM ASX and 400 μM CPF simultaneously for 24 h.

After the incubation periods (at 37 °C in a humidified 5% CO_2_), cell viability was assessed using the MTT [3-(4,5-dimethyl-2-thiazolyl)-2,5-diphenyl-2H-tetrazolium bromide] assay. Briefly, 10 μL of MTT solution was added to each well and incubated for 3 h at 37 °C according to the manufacturer’s instructions (Cayman Chemical Company, Ann Arbor, MI, USA), and then the plates were measured at 570 nm using an ELISA plate reader (BMG Labtech, Ortenberg, Germany). Cellular viability was expressed as a percentage compared to the control (control survival).

After treatment, the treated A549 cells were washed twice with PBS and fixed with 4% paraformaldehyde (PFA) solution for 15 min at room temperature. After fixation, the cells were washed again with PBS and permeabilized with PBS containing 0.1% Triton X-100 for 5 min. The cells were then incubated with 1 µg/mL 4′, 6′-diamidino-2-phenylindole (DAPI, Sigma-Aldrich, St. Louis, MO, USA) solution in the dark for 5 min. After staining, the cells were washed with PBS and mounted onto slides. The cells on the samples were evaluated using a fluorescence microscope (TE-2000; Nikon, Tokyo, Japan). DAPI-stained nuclei were quantified using ImageJ software (Version 1.53k, National Institutes of Health, Bethesda, MD, USA). For each treatment group, five randomly selected non-overlapping fields per well were analyzed, with at least 300 nuclei counted in total from three independent experiments (n = 3). Data were expressed as the percentage of intact nuclei relative to the total nuclei in each field.

To determine the appropriate CPF concentration, A549 cells were exposed to a range of CPF (CAS 2921-88-2; Sigma-Aldrich, MO, USA) concentrations (5–1000 μM) for 24 h. Cell viability was assessed, and a concentration-dependent decrease in viability was observed. Based on the results and supporting literature, 400 μM CPF reduced cell viability by approximately 50%, and this concentration was used for subsequent experiments (Figure S1). This choice is also supported by multiple in vitro studies that have utilized CPF concentrations within experimentally relevant ranges to investigate cellular toxicity mechanisms, including oxidative stress, mitochondrial dysfunction, and apoptosis, with these concentrations consistently inducing measurable and reproducible biochemical responses across various cell models, thereby providing valuable mechanistic insights into CPF toxicity [28,29]. Similarly, ASX (SML0982; Sigma-Aldrich, MO, USA) was tested at concentrations of 0.5, 5, 10, 20, 50, and 100 μM. No cytotoxic effect was observed at concentrations up to 20 μM, which was therefore chosen as the non-toxic effective dose for further studies. Each treatment group was tested in three independent biological replicates, with technical triplicates within each assay. The concentrations determined in this preliminary assessment were subsequently applied in all experimental assays described below (Table 1).

### 2.2. Lactate Dehydrogenase (LDH) Release Assay

LDH is a cytosolic enzyme that is released into the extracellular medium upon cell damage and serves as a marker for cytotoxicity. LDH catalyzes the conversion of lactate to pyruvate, reducing nicotinamide adenine dinucleotide (NAD^+^) to its reduced form.

A549 cells were treated with compounds. After treatment, the LDH cytotoxicity assay was performed based on the supplier’s instructions (Cat. No. ab102526, Abcam, Cambridge, UK). Briefly, 100 μL of supernatant was transferred to a new 96-well plate, followed by the addition of 100 μL of the LDH reaction solution to each well. After incubation for 30 min, the absorbance was measured at 490 nm using an ELISA plate reader. The amount of LDH was expressed as a percentage compared to the total amount of LDH in the cells treated with 2 % Triton-X100. The analysis was performed on three independent biological replicates (n = 3). The untreated group served as a negative control, and hydrogen peroxide (H_2_O_2_; 2.5 × 10^−5^ M) was used as a positive control for 48 h.

### 2.3. TAC, TOS, and IROX Activities

TAC and TOS were evaluated using a commercially available kit (Rel Assay Diagnostics^®^, Gaziantep, Turkey) according to the manufacturer’s instructions. A549 cells were treated with ASX and CPF in accordance with the experimental groups by a 2 h incubation at 37 °C under a humidified atmosphere of 5% CO_2_. The TAC assay measures the ability of antioxidants in the culture medium to reduce the 2,2′-azino-bis (3-ethylbenzothiazoline-6-sulfonic acid) radical. In this procedure, 30 μL of the sample was added to 500 μL of reagent 1 in a quartz cuvette. The absorbance was first measured at 660 nm after 30 s. Then 75 μL of reagent 2 was added and the absorbance was measured at 660 nm after 5 min. Results were calibrated against Trolox and expressed as mmol Trolox equivalents per liter. Ascorbic acid (10^−5^ M) was used as a positive control. The analysis was performed on three independent biological replicates (n = 3).

The TOS assay quantifies oxidizing agents based on their ability to convert an iron (II) ion chelator complex to iron (III) ions. Here, 500 μL of reagent 1 was mixed with 75 μL of the sample and the absorbance was measured after 30 s at 530 nm. Then 15 μL of reagent 2 was added and another measurement was performed at 530 nm. The results were calibrated against H_2_O_2_ (2.5 × 10^−5^ M) as a positive control for 48 h and expressed as μM H_2_O_2_ equivalents per liter. A total of 50 metaphase plates, which showed various chromosomal abnormalities, were analyzed and enumerated for further evaluation. The analysis was performed on three independent biological replicates (n = 3).

Intracellular reactive oxygen species production was evaluated using CellROX^®^ Oxidative Stress Reagent (Cat. No. C10444, Thermo Fisher Scientific, Waltham, MA, USA). A549 cells were seeded into ultra-low attachment 24-well plates at a density of 1 × 10^5^ cells/well and incubated for 24 h. Cells were then treated with ASX and CPF in accordance with the experimental groups. Thirty minutes prior to the end of the treatment period, CellROX^®^ Green was added at a final concentration of 5 μM and incubated at 37 °C in the dark. After incubation, cells were gently collected by pipetting and washed twice with PBS to remove excess dye. Fluorescence images were captured using a confocal microscope (ZEISS LSM 800). As a positive control, cells treated with H_2_O_2_ (2.5 × 10^−5^ M) were included to validate ROS induction. All assays were performed in triplicate. The mean fluorescence intensity (MFI) was used as a quantitative indicator of intracellular ROS levels. The analysis was performed on three independent biological replicates (n = 3).

### 2.4. Measurement of MMP (ΔΨm)

The MMP (ΔΨm) was assessed using a quenching-mode Safranin O assay, as previously described with modifications [30]. A549 cells were seeded in 6-well plates and treated under the following experimental conditions: control, CPF (400 µM), ASX (20 µM), pretreatment (ASX 6 h + CPF 24 h), and co-treatment (ASX + CPF for 24 h). After treatment, cells were collected, washed with PBS, and mitochondria were isolated using the MITOISO1 mitochondrial isolation kit (Cat. No. MITOISO1, Sigma-Aldrich, St. Louis, MO, USA) according to the manufacturer’s instructions.

The protein concentration of the mitochondrial fractions was determined using the Bradford assay. For fluorescence measurement, 25 μg of mitochondrial protein was mixed with 0.04 mM Safranin O in a final volume of 100 μL. Fluorescence was measured using a BioTek Synergy H1 microplate reader (Agilent Technologies, Santa Clara, CA, USA) at 495 nm excitation and 586 nm emission at 37 °C. Fluorescence intensity was recorded and normalized to the control group. Since Safranin O operates in a quenching mode, an increase in fluorescence indicates a decrease in ΔΨm, i.e., mitochondrial depolarization. MMP was quantified as total MFI using a fluorescence microplate reader (Ex/Em: 549/574 nm). All experiments were performed in triplicate, and results were expressed as percentage fluorescence relative to the control group.

### 2.5. GPx Activity Assay

GPx is a critical enzyme in the antioxidant defense system of cells, protecting against oxidative stress by reducing hydrogen peroxide and lipid peroxides. According to the manufacturer’s instructions, the Glutathione Peroxidase Assay Kit (Abcam, Cat No. ab102530) was used to measure the enzymatic activity of GPx in treated A549 cells. The assay is based on a coupled enzyme reaction, which involves the reduction of a substrate by GPx in the presence of NADPH. The oxidation of NADPH results in a decrease in absorbance at 340 nm, which is directly proportional to the GPx activity in the sample. The analysis was performed on three independent biological replicates (n = 3). The untreated group served as the negative control, and CPF-treated cells served as the positive control. Results are expressed as units per milligram of protein (U/mg protein).

### 2.6. MDA Activity Assay

Lipid peroxides were detected as MDA, which reacts with thiobarbituric acid (TBA) (Abcam, Cambridge, UK) to form an MDA-TBA adduct that can be measured by colorimetric analysis. Briefly, the treated cells were pelleted and lysed with MDA lysis buffer and 200 μL of the supernatant was collected by centrifugation at 10,000× *g* for 10 min at 4 °C. The supernatant was used to quantify the lipid peroxides using the TBA assay. Six hundred microliters of TBA solution were added to the collected supernatant and incubated at 95 °C for 60 min. The mixture was then cooled for 10 min at room temperature in an ice bath. Finally, 200 μL of the mixture was added to a 96-well microplate for colorimetric analysis. The absorbance was measured at 532 nm using a Victor™ X3 multilabel reader (PerkinElmer, Waltham, MA, USA). The standard curve showed excellent linearity (R^2^ = 0.998) within a concentration range of 0.1–10 nmol/mL. The limit of detection (LOD) was calculated as 0.08 nmol/mL, and the limit of quantification (LOQ) was 0.15 nmol/mL. MDA concentrations were calculated using MDA as a reference standard, with TBA amounts expressed in nmol per mg protein. The analysis was performed on three independent biological replicates (n = 3). Untreated group served as negative control, and CPF-treated cells served as positive control.

### 2.7. SOD Activity Assay

A commercially available superoxide dismutase activity kit (Cat. No. ab65354, Abcam®, Cambridge, UK) was used to measure SOD activity. Briefly, the treated cells were pelleted and lysed with a lysis buffer (0.1 M Tris/HCl, 0.5% Triton X-100, 5 mM β-ME, 0.1 mg/mL) on ice for 10 min. To remove insoluble substances, the samples were centrifuged at 10,000× *g* for 10 min at 4 °C and the supernatant was transferred to fresh 48-well plates. For analysis, 20 μL of the sample and 20 μL of the blank (dH_2_O) were mixed with 200 μL of the WST working solution. Subsequently, 20 μL of the enzyme working solution was added to each sample and incubated at 37 °C for 20 min. The color intensities were read at 450 nm using a microplate reader. The analysis was performed on three independent biological replicates (n = 3). Untreated groups served as negative control, and CPF-treated cells served as positive control. Results are expressed as units per milligram of protein (U/mg protein).

### 2.8. Measurement of DNA Fragmentation

DNA fragmentation was quantified using the diphenylamine (DPA) colorimetric assay, based on the protocol of Burton [30] with minor modifications. Briefly, A549 cells (1 × 10^5^ cells/well) were seeded in 6-well plates and treated according to the experimental design. After treatment, cells were harvested and lysed in hypotonic lysis buffer (10 mM Tris-HCl, 10 mM EDTA, 0.5% Triton X-100, pH 7.4), followed by centrifugation at 13,000× *g* for 20 min at 4 °C to separate DNA (supernatant) from intact chromatin (pellet). Both fractions were precipitated overnight at 4 °C with 12.5% trichloroacetic acid (TCA) and then hydrolyzed in 5% TCA at 90 °C for 15 min. After cooling, 1 mL of freshly prepared (DPA) reagent (150 mg DPA in 10 mL glacial acetic acid with 150 µL of concentrated sulfuric acid and 50 µL of 1.6% acetaldehyde solution) was added to each tube. Samples were incubated at 37 °C for 4 h, and the color intensity was measured spectrophotometrically at 600 nm.

The percentage of fragmented DNA was calculated using the formula:

% DNA fragmentation = [OD_600_ (supernatant)/(OD_600_ supernatant + pellet)] × 100

### 2.9. Intracellular Ca^2+^ Measurement Using ICP-OES

Intracellular Ca^2+^ measurement was performed according to the method modified by Güner et al. [31]. A549 cells were seeded at a density of 1 × 10^6^ cells/well in 6-well culture plates and allowed to adhere for 24 h at 37 °C in a humidified 5% CO_2_ incubator. The cells were divided into five groups: untreated control, CPF alone (400 μM), ASX alone (20 μM), pretreatment group (ASX 20 μM for 6 h followed by CPF 400 μM for 24 h), and co-treatment group (ASX 20 μM + CPF 400 μM simultaneously for 24 h). After treatment, the cells were washed twice with cold PBS to remove extracellular calcium. The cells were then collected and resuspended in 3 mL of ultrapure nitric acid (65%, trace metal grade), and digested at 95 °C on a heating block until the solution became clear and colorless. After cooling, the samples were diluted in 0.2% nitric acid and filtered using a 0.22 μm membrane filter. Intracellular Ca^2+^ concentrations were determined using Inductively Coupled Plasma–Optical Emission Spectrometry (ICP-OES) (Thermo Scientific iCAP™ Series, Thermo Fisher Scientific, Waltham, MA, USA). Calibration curves were prepared by analyzing a series of Ca^2+^ standard solutions (Ca^2+^ standard solutions traceable to NIST, prepared in 0.2% nitric acid) covering the concentration range of 1–100 ng/mL. The calibration curve showed excellent linearity with a coefficient of determination (R^2^) of 0.996, with a linear range of 1–250 ng/10^6^ cells. The limit of detection (LOD) and limit of quantification (LOQ) were 0.9 ng/10^6^ cells and 1.8 ng/10^6^ cells, respectively, based on the standard deviation of the blank and the slope of the calibration curve. Ca^2+^ levels were expressed as ng/10^6^ cells. All measurements were performed in triplicate. Untreated cells were used as the baseline control for intracellular Ca^2+^ content.

### 2.10. Quantitative Real-Time Polymerase Chain Reaction (RT-PCR)

The effects of different treatments—control, CPF (400 µM), ASX (20 µM), pretreatment (ASX 6 h + CPF 24 h), and co-treatment (ASX + CPF, 24 h)—on the expression of genes related to antioxidant/stress response (Nrf2, HO-1), inflammation (IL-6, IL-1β, TNF-α, NFκB [RELA subunit]), and apoptosis/cell death (Caspase-3, TP53, BAX, BCL-2, VDAC1) were evaluated by RT-qPCR analysis. Briefly, A549 cells treated with compounds were harvested, and total RNA was isolated using the TriPure isolation reagent (Roche, Basel, Switzerland, Cat. no. 11 667 157 001). The quality of the isolated RNA was controlled by NanoDrop (NanoDrop ND-2000c, Thermo Scientific, Waltham, MA, USA). First strand cDNA was synthesized from total RNA with the Transcriptor First Strand cDNA Synthesis kit (Cat. No. 04 379 012 001, Roche, Basel, Switzerland). Real-time polymerase chain reaction (RT-PCR) analysis was conducted on the LightCycler v.1.5 instruments (Roche Applied Science, Basel, Switzerland) and performed with SYBR Green PCR Master Mix (Cat. No. 204143, Qiagen, Hilden, Germany). The RT-PCR reaction was performed in a 10 µL mixture containing 5 μL SYBR Green PCR Master Mix, 0.5 μL cDNA, and 0.3 μM primers. Cycling conditions for the PCR reaction were as follows: initially 10 min at 95 °C, followed by 40 cycles of cyclic denaturation at 95 °C for 15 s, annealing at 59 °C for 1 min, and extension for 13 s at 72 °C. β-actin was used as the internal reference gene in the qPCR analyses. The stability of β-actin expression was confirmed by observing similar Ct values across all experimental groups (intergroup difference < 0.5 cycles) and single-peaked melting curve profiles, indicating consistent reference gene expression throughout the study. The comparative threshold cycle (Ct) method (2^−∆∆Ct^ method) was used to determine the expression level of the analyzed genes. Ct values were determined at the same fluorescence threshold line for all the genes, and the Ct values for each sample were obtained by calculating the arithmetic mean of triplicate values. PCR was performed with specific primers of the genes (Table 2).

**Table 2:** Accession number and specific primers of the genes.

Gene	Accession Number	Primers
BAX	NM_138764	F-GAGTGTCTCAAGCGCATCG
		R-GCAAAGTAGAAAAGGGCGACA
TNF	NM_000594	F-CTCTTCTGCCTGCTGCACTTTG
		R-ATGGGCTACAGGCTTGTCACTC
Bcl2	NM_000633	F-GTGGCCTTCTTTGAGTTCGG
		R-GGCCGTACAGTTCCACAAA
Caspase-3	NM_004346	F-AGCGAATCAATGGACTCTGGA
		R-GGTTTGCTGCATCGACATCT
HO-1	NM_002133	F-CTTTTCCTCAGGAGTTCCGC
		R-CAAACAGCTCCTGCAACTTG
TP53	NM_000546	F-GTCCAGATGAAGCTCCCAGA
		R-CAAGAAGCCCAGACGGAAAC
RELA (NF-κB p65)	NM_021975	F-GAAAGGACTTCCAAGATTGGG
		R-GGGCTGCAGATCTCCTTTGC
NRF2	NM_006164	F-CACATCCAGTCAGAAACCAGTGG
		R-GGAATGTCTGCGCCAAAAGCTG
IL-6	NM_000600	F-TGGACTACCTGCACTCGGAGAA
		R-GTGCCGCAAAAGGTCTTCATGG
IL1B	NM_000576	F-TGGCGGCATCCAGCTACGAA
		R-CCGGAGCGTGCAGTTCAGTGA
VDAC1	NM_003374	F-TGGCTGTGTCCTGTGTTGAT
		R-GGTGTTCTCGGGTAGTTTGC
β-actin	NM_001101	F-CTCCATCCTGGCCTCGCTGT
		R-GCTGTCACCTTCACCGTTCC

### 2.11. In Ovo HET-CAM (Hen’s Egg Test Chorioallantoic Membrane) Test

The HET-CAM assay was performed to evaluate the angiogenic or anti-angiogenic effects of CPF, ASX, and their combination. The possible effects of the samples were tested on fertilized Leghorn chicken eggs weighing 50–60 g from commercial sources using the HET-CAM method modified according to Güner and Karabay Yavaşoğlu [32]. The fertilized chicken eggs were placed in an incubator with a conveyor belt rotation system at 37  ±  1 °C and 80  ±  2% humidity for 7 days. On day 7, the eggs were opened on the stump side and aspirated through a hole on the pointed side. A round piece of the shell (3–4 cm in diameter) was then carefully removed with forceps. The inner membrane was then carefully removed with forceps without damaging the blood vessels. Then, sterile filter paper discs (5 mm diameter) were impregnated with test compounds and placed directly on the CAM surface near visible blood vessels. The following treatment groups were established (n = 6 eggs per group):

Control group: 50 µL of vehicle solution (0.9% NaCl)

Positive control: 50 µL of Dexamethasone (100 µg/mL; ~5 µg)

CPF group: 50 µL of CPF solution (400 µM; ~7 µg)

ASX group: 50 µL of ASX solution (20 µM; ~0.6 µg)

Co-treatment: 50 µL containing CPF (400 µM) + ASX (20 µM) mixed in a single solution

Pretreatment: 50 µL of ASX (20 µM) was placed onto the CAM surface and incubated for 1 h at 37.5 °C. After incubation, the disc was gently removed, and a new disc containing 20 µL of CPF (400 µM) was applied to the same area.

All compounds were freshly prepared using DMSO as the initial solvent and diluted with PBS to yield a final DMSO concentration of ≤0.1% (*v*/*v*), ensuring minimal solvent-related irritation. The discs were gently placed on the CAM using sterile forceps, and the eggs were sealed and incubated for an additional 48 h. After the incubation, eggs were photographed. Angiogenesis scores and anti-angiogenic effects of the compounds on CAM were assessed as follows:

Average score = [number of eggs (score 2) × 2 + number of eggs (score 1) × 1]/[total number of eggs (score 0, 1, 2)].

According to this system, a score <0.5 indicated that there was no anti-angiogenic effect, 0.5–1 indicated a low anti-angiogenic effect, and >1 indicated a powerful anti-angiogenic effect.

Briefly, <0.5, No effect: normal embryo development. There is no change according to the surrounding capillaries. No hemorrhage, vascular lysis, or coagulation was detected.

0.5–1, Low: The area without the covers is low or the density of the capillary is reduced in a specific area. The effects are not more than 2 times the field of matter.

>1, High: There is space without capillaries. Normal embryo formation is not observed.

All samples were tested in triplicate at different time points.

### 2.12. Statistical Analysis

Statistical analysis was performed using SPSS 20.0 (SPSS, Chicago, IL, USA). Differences between groups were assessed using one-way analysis of variance (ANOVA) followed by Tukey’s post hoc test for multiple comparisons. All experiments were performed in triplicate and data were expressed as the means ± standard deviation. A *p*-value less than 0.05 (≤0.05) was considered statistically significant.

## 3. Results

### 3.1. Cell Viability

**Figure 1:**
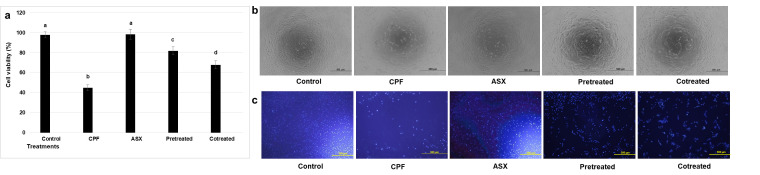
Effect of different treatments on cell viability (%) (**a**); morphological changes (**b**,**c**); fluorescence photomicrographs of DAPI staining (blue) in A549 cells (scale bars: 500 μM). A549 cells were exposed to ASX (20 μM), CPF (400 μM), pretreatment (ASX for 6 h followed by CPF for 24 h), and co-treatment (ASX + CPF for 24 h) by MTT assay. Values are the mean ± SD of three experiments and are expressed as percentage with respect to the untreated control. Statistical analysis was performed using one-way ANOVA followed by Tukey’s post hoc test. Bars indicated by different letters show significant differences between groups: CPF compared to the control, *p* = 0.00016; pretreatment compared to the CPF, *p* = 0.0011; co-treatment compared to the CPF, *p* = 0.0038. ASX: Astaxanthin, CPF: Chlorpyrifos.

CPF treatment significantly reduced cell viability compared with the control group, with a fold decrease of 2.17 (*p* = 0.00016). ASX alone did not significantly alter viability (*p* = 0.673 compared with control). Pretreatment with ASX prior to CPF exposure significantly improved cell viability by 1.82-fold compared with CPF-treated cells (*p* = 0.0011). Co-treatment with ASX and CPF increased viability by 1.51-fold compared with CPF-treated cells (*p* = 0.0038) (Figure 1a,b).

Consistent with the MTT assay results, DAPI staining showed that CPF exposure significantly reduced the proportion of morphologically intact nuclei to 47.6 ± 4.1% of control values (*p* = 0.00085). The pretreatment (78.2 ± 3.7%, *p* = 0.0012) and co-treatments (70.5 ± 4.4%, *p* = 0.0045) also preserved the nuclear appearance and increased the number of nuclei compared to CPF treatment, especially in the pretreatment group (Figure 1c).

### 3.2. LDH, TAC, TOS, and IROS Activities

**Figure 2:**
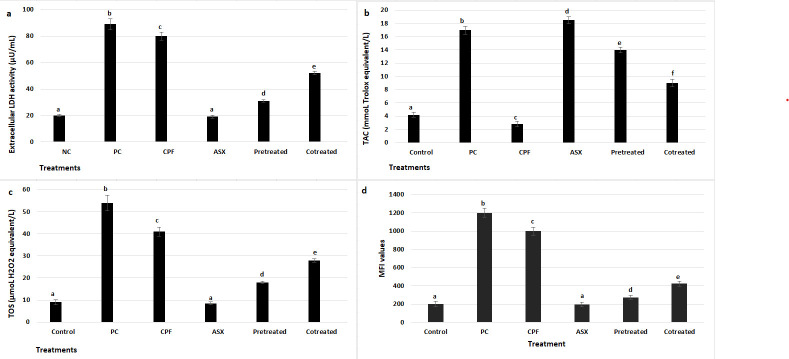
Extracellular LDH activity (µU/mL) (**a**), total antioxidant status (TAC) (**b**), total oxidative stress (TOS) (**c**), and intracellular ROS levels (IROS) (MFI) (**d**) after treatment with ASX (20 μM), CPF (400 μM), pretreatment (ASX for 6 h followed by CPF for 24 h), and co-treatment (ASX + CPF for 24 h) in A549 cells. LDH activity was measured to evaluate membrane damage and cytotoxicity, while TAC and TOS assays were used to assess oxidative balance. Intracellular ROS levels were determined using the CellROX^®^ Green reagent. Data represent means ± SD of at least three experiments. Statistical analysis was performed using one-way ANOVA followed by Tukey’s post hoc test. Bars indicated by different letters show significant differences between groups: (CPF compared to the control; *p* = 0.00095 for LDH; *p* = 0.0434 for TAC; *p* = 0.0004 for TOS; *p* = 0.000066 for IROS) (Pretreatment compared to the CPF; *p* = 0.001185 for LDH; *p* = 0.0035 for TAC; *p* = 0.000264 for TOS; *p* = 0.000133 for IROS) (Co-treatment compared to the CPF; *p* = 0.003909 for LDH; *p* = 0.00806 for TAC; *p* = 0.001029 for TOS; *p* = 0.000846 for IROS). PC: Positive control as ascorbic acid (10^−5^ M) for TAC and H_2_O_2_ (2.5 × 10^−5^ M) for TOS, LDH, and IROS; ASX: Astaxanthin, CPF: Chlorpyrifos.

To further assess cytotoxic effects, LDH release was measured as an indicator of membrane damage (Figure 2a). CPF exposure caused a marked increase in LDH release, with a 4-fold elevation compared to the control group (*p* = 0.00095). ASX alone had no significant effect on LDH levels (*p* = 0.0642). ASX pretreatment significantly reduced LDH release compared to CPF alone, with a 2.58-fold decrease (*p* = 0.001185), while co-treatment also attenuated LDH elevation by 1.54-fold (*p* = 0.003909).

The changes in TAC levels following treatment with CPF, ASX, and their pretreatment and co-treatment were presented in Figure 2b. CPF significantly reduced TAC levels by 1.5-fold compared to the control group (*p* = 0.0434). In contrast, ASX treatment alone markedly increased TAC by 4.4-fold compared to the control (*p* = 0.000142). Pretreatment with ASX prior to CPF exposure also significantly elevated TAC levels (3.33-fold increase) (*p* = 0.0035), while co-treatment resulted in a 1.93-fold increase in TAC levels compared to the CPF group (*p* = 0.00806).

TOS levels were also measured to assess oxidative stress after treatments (Figure 2c). CPF significantly increased TOS levels by 4.56-fold compared to the control group (*p* = 0.0004), indicating a high level of oxidative stress. ASX alone did not change TOS levels. Both pretreatment and co-treatment with ASX decreased TOS levels by 2.27-fold and 1.46-fold, respectively, compared to CPF alone (*p* = 0.000264) and (*p* = 0.001029).

IROS levels were assessed using CellROX staining (Figure 2d). CPF exposure markedly increased ROS production, showing a 5-fold increase compared to the control group (*p* = 0.000066), while ASX alone did not affect the ROS levels (*p* = 0.0764). Pretreatment significantly reduced the ROS levels with a 3.64-fold decrease compared to CPF alone (*p* = 0.000133). Co-treatment significantly diminished ROS production with a 2.35-fold change compared to CPF alone (*p* = 0.000846).

### 3.3. Mitochondrial Membrane Potential (MMP) Changes

**Figure 3:**
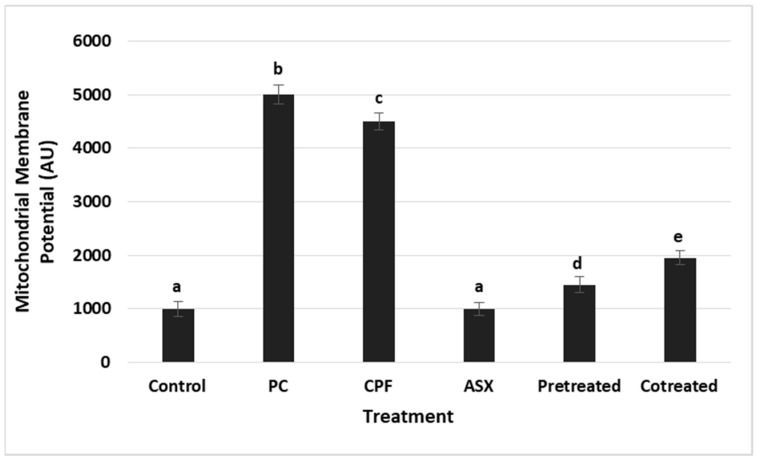
Quantitative analysis of mitochondrial membrane potential (MMP) after treatment with ASX (20 μM), CPF (400 μM), pretreatment (ASX for 6 h followed by CPF for 24 h), and co-treatment (ASX + CPF for 24 h) in A549 cells. MMP levels were measured using a fluorescent probe and are expressed as arbitrary fluorescence units (AU). Data represent means ± SD of at least three experiments. Statistical analysis was performed using one-way ANOVA followed by Tukey’s post hoc test. Bars indicated by different letters show significant differences: (CPF compared to the control, *p* = 0.000014) (Pretreatment compared to the CPF, *p* = 0.000201; Co-treatment compared to the CPF, *p* = 0.000538). PC: Positive control H_2_O_2_ (2.5 × 10^−5^ M) for MMP. ASX: Astaxanthin, CPF: Chlorpyrifos.

Changes in MMP were evaluated as an indicator of mitochondrial integrity (Figure 3). CPF exposure led to a significant elevation in MMP levels, representing a 4.5-fold increase compared to the control group (*p* = 0.000014), suggesting mitochondrial dysfunction. ASX alone had no significant effect on MMP. Pretreatment significantly reduced the MMP elevation with a value of 3.1-fold decrease compared to CPF alone (*p* = 0.000201). Co-treatment also significantly lowered MMP, reflecting a 2.3-fold reduction compared to CPF alone (*p* = 0.000538).

### 3.4. SOD, GPx, and MDA Levels

**Figure 4:**
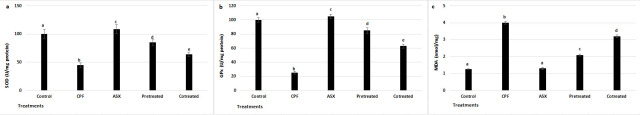
Superoxide dismutase (SOD) (**a**), glutathione peroxidase (GPx) (**b**), and malondialdehyde (MDA) (**c**) levels after treatment with ASX (20 μM), CPF (400 μM), pretreatment (ASX for 6 h followed by CPF for 24 h), and co-treatment (ASX + CPF for 24 h) in A549 cells. SOD (U/mg protein) and GPx (U/mg protein) activities were measured as antioxidant defense markers, while MDA levels (mmol/mg) were assessed as an indicator of lipid peroxidation and oxidative damage. Values represent means ± SD of at least three experiments. Statistical analysis was performed using one-way ANOVA followed by Tukey’s post hoc test. Bars indicated by different letters show significant differences: (CPF compared to the control; *p* = 0.0026 for SOD; *p* = 0.00285 for GPx; *p* = 0.0019 for MDA) (pretreatment compared to the CPF; *p* = 0.0048 for SOD, *p* = 0.00463 for GPX, *p* = 0.0021 for MDA) (co-treatment compared to the CPF; *p* = 0.0082 for SOD, *p* = 0.00782 for GPx, *p* = 0.0052 for MDA). ASX: Astaxanthin, CPF: Chlorpyrifos.

As shown in Figure 4a, CPF treatment significantly decreased SOD activity by 2.22-fold compared to the control group (*p* = 0.0026). ASX alone increased SOD activity by 1.1-fold compared to the control (*p* = 0.030706). Pretreatment resulted in a 1.88-fold increase in SOD activity compared to CPF alone (*p* = 0.0048), while co-treatment led to a 1.42-fold increase (*p* = 0.0082).

As illustrated in Figure 4b, GPx activity was also markedly suppressed by CPF treatment with a value of 4-fold change compared to control (*p* = 0.00285). ASX alone enhanced GPx activity by 1.05-fold compared to control (*p* = 0.0261). Pretreatment with ASX significantly increased GPx levels by 3.4-fold compared to CPF (*p* = 0.00463), while co-treatment led to a 2.52-fold increase (*p* = 0.00782).

To assess lipid peroxidation, MDA levels were measured (Figure 4c). CPF exposure resulted in a 3.15-fold increase in MDA levels compared to control (*p* = 0.0019), indicating oxidative damage to membrane lipids. ASX alone showed no significant difference in MDA compared to control (*p* = 0.0806). However, ASX pretreatment significantly reduced MDA levels by 1.90-fold (*p* = 0.0021), while co-treatment reduced MDA by 1.25-fold (*p* = 0.0052), both compared to CPF.

### 3.5. Intracellular Ca^2+^ Levels

**Figure 5:**
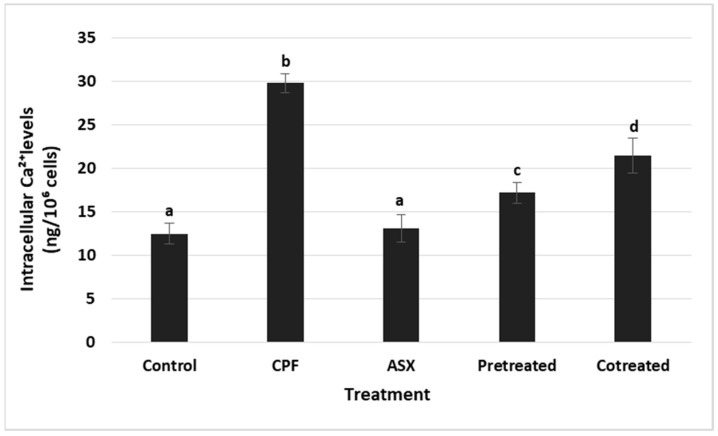
Changes in intracellular Ca^2+^ concentrations (ng/10^6^ cells) in A549 exposed to ASX (20 μM), CPF (400 μM), pretreatment (ASX for 6 h followed by CPF for 24 h), and co-treatment (ASX + CPF for 24 h). Values represent means ± SD of at least three experiments. Statistical analysis was performed using one-way ANOVA followed by Tukey’s post hoc test. Bars indicated by different letters show significant differences: (CPF compared to the control, *p* = 0.00171) (pretreatment compared to the CPF, *p* = 0.0038; co-treatment compared to the CPF, *p* = 0.0063). ASX: Astaxanthin, CPF: Chlorpyrifos.

Intracellular Ca^2+^ levels were measured to assess calcium homeostasis under CPF-induced stress conditions (Figure 5). CPF exposure resulted in a significant increase in Ca^2+^ levels compared to the control (*p* = 0.00171). ASX alone did not significantly alter intracellular Ca^2+^ levels compared to the control group (*p* = 0.0742). Pretreatment with ASX significantly increased the CPF-induced calcium elevation with a value of 1.73-fold change (*p* = 0.0038). Co-treatment also resulted in a reduction, with a value of 1.39-fold decrease compared to the CPF (*p* = 0.0063).

### 3.6. DNA Fragmentation

**Figure 6:**
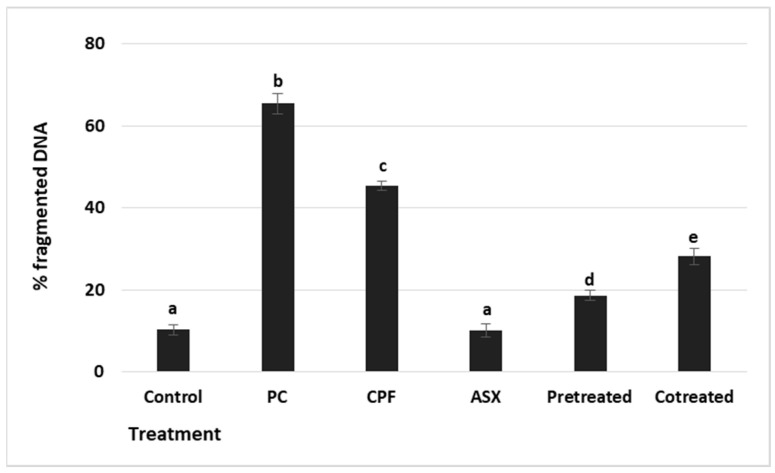
DNA fragmentation rate (%) in A549 exposed to ASX (20 μM), CPF (400 μM), pretreatment (ASX for 6 h followed by CPF for 24 h), and co-treatment (ASX + CPF for 24 h). The diphenylamine assay was used for the evaluation of the content of fragmented DNA. Values represent means ± SD of at least three experiments. Statistical analysis was performed using one-way ANOVA followed by Tukey’s post hoc test. Bars indicated by different letters show significant differences: (CPF increased compared to the control, *p* = 0.000183) (pretreatment compared to the CPF, *p* = 0.00105; co-treatment compared to the CPF, *p* = 0.00504). PC: Positive control H_2_O_2_ (2.5 × 10^−5^ M) for DNA fragmentation. ASX: Astaxanthin, CPF: Chlorpyrifos.

To evaluate cytotoxicity-associated DNA damage, DNA fragmentation was measured by the diphenylamine (DPA) colorimetric assay (Figure 6). The positive control showed a maximal increase (6.00-fold) (*p* = 0.000036), confirming the assay’s sensitivity. CPF exposure led to a significant increase in DNA fragmentation with a value of 4.4-fold compared to the control group (*p* = 0.000183). ASX alone did not affect DNA fragmentation levels compared to the control (*p* = 0.062). Pretreatment significantly reduced DNA fragmentation by a 2.3-fold change compared to CPF alone (*p* = 0.00105), while co-treatment resulted in a 1.56-fold reduction (*p* = 0.00504).

### 3.7. Gene Expression Levels

**Figure 7:**
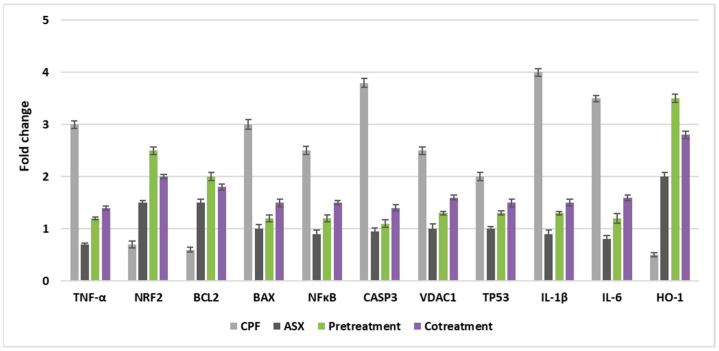
Fold changes in gene expression levels in A549 exposed to ASX (20 μM), CPF (400 μM), pretreatment (ASX for 6 h followed by CPF for 24 h), and co-treatment (ASX + CPF for 24 h) using real-time quantitative PCR (RT-qPCR). Relative expressions were calculated using the 2^−∆∆Ct^ method and normalized to β-actin as an internal control. Values represent means ± SD of at least three experiments. ASX: Astaxanthin, CPF: Chlorpyrifos.

As shown in Figure 7, the expression levels of genes associated with antioxidant defense (Nrf2, HO-1), inflammation (IL-6, IL-1β, TNF-α, NFκB), and apoptosis (Caspase-3, TP53, BAX, BCL-2, VDAC1) were significantly altered by pre- and co-treatment with ASX in CPF-induced lung cells.

CPF treatment led to a downregulation of Nrf2 expression (0.7-fold), indicating a suppression of the antioxidant response. In contrast, ASX alone significantly upregulated Nrf2 with a value of 1.5-fold change. Pretreatment with ASX prior to CPF exposure enhanced Nrf2 expression markedly with a value of 2.5-fold change, while co-treatment also showed a notable upregulation with a value of 2.0-fold change, suggesting that ASX mitigates CPF-induced oxidative stress by activating the Nrf2 pathway.

HO-1 expression was downregulated by CPF (0.5-fold). ASX alone upregulated HO-1 levels with a value of 2.0-fold change, while pretreatment significantly upregulated with a value of 3.5-fold change. Co-treatment also upregulated HO-1 expression with a value of 2.8-fold change. These findings support the role of ASX in activating the Nrf2/HO-1 antioxidant axis against CPF toxicity.

CPF significantly induced IL-6 expression with a value of 3.5-fold change, reflecting a strong pro-inflammatory response. ASX alone downregulated IL-6 levels (0.8-fold change). Pretreatment and co-treatment significantly downregulated the CPF-induced IL-6 expression, with values of 1.2-fold change and 1.6-fold change, respectively, indicating the anti-inflammatory effects of ASX.

IL-1β levels were upregulated by CPF treatment with a value of 4-fold change. Pretreatment and co-treatment significantly downregulated these upregulations, with values of 1.3-fold change and 1.5-fold change, suggesting ASX can suppress CPF-induced pro-inflammatory cytokine production.

CPF resulted in a 3-fold increase in TNF-α expression. ASX alone downregulated TNF-α (0.7-fold). Pretreatment with ASX downregulated the CPF-induced elevation with a value of 1.1-fold change, while co-treatment also decreased expression with a value of 1.4-fold change, supporting the anti-inflammatory role of ASX.

CPF caused a 2.5-fold increase in RELA (NFκB subunit) expression. Pretreatment and co-treatment reduced RELA levels to 1.2-fold change and 1.5-fold change, respectively, indicating inhibition of the NFκB inflammatory pathway by ASX.

CPF significantly upregulated Caspase-3 (3.8-fold), indicating activation of apoptotic signaling. ASX did not significantly change. Both pretreatment (1.1-fold change) and co-treatment (1.4-fold change) downregulated the CPF-induced increase, suggesting that ASX mitigates CPF-induced apoptosis.

TP53 expressions were upregulated by CPF treatment with a value of 2.0-fold change. ASX had no significant effect. ASX pretreatment (1.3-fold change) and co-treatment (1.5-fold change) downregulated the CPF-induced upregulation, reflecting potential regulation of the DNA damage response.

CPF induced a 3-fold increase in BAX expression, consistent with pro-apoptotic activation. ASX alone did not cause a significant change in BAX expression, but pretreatment (1.2-fold change) and co-treatment (1.5-fold change) downregulated CPF-induced upregulations, indicating inhibition of the apoptotic pathway.

CPF downregulated anti-apoptotic BCL-2 expression (0.6-fold change). ASX significantly increased BCL-2 (1.5-fold change). Pretreatment and co-treatment upregulated BCL-2 levels, with values of 2.0-fold change and 1.8-fold change, respectively, demonstrating ASX’s anti-apoptotic potential.

VDAC1 expressions were upregulated by CPF with a value of 2.5-fold change, suggesting mitochondrial membrane destabilization. ASX alone did not cause a significant change in VDAC1 expression. Pretreatment and co-treatment downregulated the CPF-induced VDAC1 expression with values of 1.3-fold change and 1.6-fold change, respectively, supporting the mitochondrial protective effects of ASX.

### 3.8. In Ovo HET-CAM Assay

**Figure 8:**
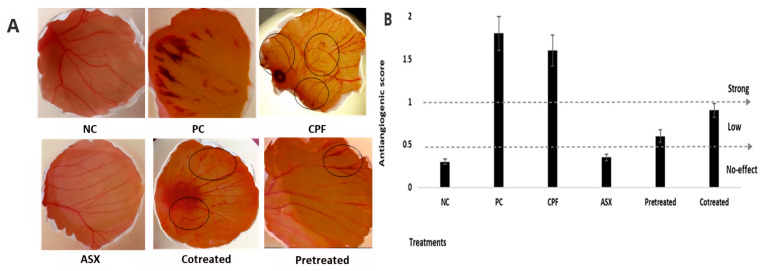
HET-CAM assay. (**A**) Pictures indicating how various endpoints on membrane following ASX, CPF, and their combination treatments. (**B**) Anti-angiogenic scores after treatments of ASX, CPF, and their combination on membrane surface. Values represent means ± SD of at least three experiments. ASX: Astaxanthin, CPF: Chlorpyrifos, NC: 0.9% NaCl was tested as negative control. PC: Dexamethasone (100 µg/mL) was tested as positive control.

To evaluate the potential anti-angiogenic effects of ASX against CPF-induced vascular alterations, a CAM assay was performed, and angiogenesis was semi-quantitatively scored. Angiogenic responses were classified based on the number and complexity of blood vessels around the treatment area, and scores were assigned as previously described. CPF treatment (400 µM) induced a significant increase in neovascularization, with a mean angiogenesis score of 1.62 ± 0.18, indicating a strong pro-angiogenic response compared to the control group (0.3 ± 0.04). In contrast, ASX alone (20 µM) did not significantly alter vascular structure, with a mean angiogenesis score of 0.35 ± 0.08, similar to the control group, suggesting no intrinsic angiogenic or anti-angiogenic activity under physiological conditions. Co-treatment resulted in a marked reduction in angiogenesis compared to CPF alone (0.95 ± 0.08). Similarly, pretreatment application led to a more pronounced anti-angiogenic response, with a significantly lower score of 0.65 ± 0.07, suggesting a stronger protective effect compared to co-treatment (Figure 8).

## 4. Discussion

This study comprehensively evaluated the protective effect of ASX against CPF-induced toxicity in A549 lung epithelial cells using pre- and co-treatment strategies. CPF, a widely used organophosphate pesticide, is well known for its ability to induce oxidative stress through excessive ROS generation. This damage compromises mitochondrial activity, DNA integrity, and membrane stability, particularly in lung tissues, where it can contribute to pulmonary fibrosis and acute respiratory distress syndrome [33]. Our findings demonstrate that ASX exerted significant protective effects, especially when administered as pretreatment, by modulating oxidative, inflammatory, apoptotic, and mitochondrial pathways.

CPF exposure markedly reduced cell viability in A549 cells, primarily via oxidative stress and subsequent cellular damage. Previous studies on neuroblastoma, kidney, and liver cells have also reported that CPF decreases cell viability by inducing oxidative stress and mitochondrial dysfunction in a concentration- and time-dependent manner [33,34,35]. Consistent with these reports, we observed CPF-induced oxidative damage in lung cells, evidenced by a significant suppression of antioxidant defenses and increased ROS production [36].

Mitochondria are central targets of CPF-induced toxicity. CPF disrupts mitochondrial function, leading to depolarization of the mitochondrial membrane, excessive ROS production, and activation of apoptotic pathways [37,38]. These ROS promote lipid peroxidation, damaging membrane lipids and altering membrane integrity, which in turn disrupts protein function and increases permeability. Such damage was reflected by elevated LDH release, indicating compromised membrane integrity [39]. The heightened oxidative stress is likely linked to the inhibition of complexes I and III in the mitochondrial electron transport chain, as reported for other organophosphates [40]. In parallel, the CPF-induced decline in antioxidant enzyme activities, including GPx and mitochondrial manganese SOD, indicates depletion of both mitochondrial and cytosolic redox buffering systems [41]. Therapeutic strategies for CPF toxicity focus on reducing oxidative stress, enhancing detoxification pathways, and preserving cellular integrity. ASX effectively counteracted CPF-induced mitochondrial oxidative stress by decreasing intracellular ROS and TOS while restoring TAC in lung cells. These effects suggest that ASX not only scavenges free radicals but also stabilizes mitochondrial antioxidant systems and maintains mitochondrial function. The observed upregulation of the Nrf2/HO-1 axis supports this mechanism, as this pathway is known to enhance mitochondrial antioxidant capacity and biogenesis [22,42,43]. ASX pretreatment significantly restored the activities of SOD and GPx, both markedly suppressed by CPF exposure. This reactivation of enzymatic defenses indicates that ASX contributes to the maintenance of mitochondrial redox homeostasis, thereby reducing oxidative damage. Lower MDA levels following ASX treatment further confirm its role in protecting mitochondrial membranes from lipid peroxidation. ASX’s unique polar–nonpolar molecular structure likely facilitates its incorporation into mitochondrial membranes, enhancing stability and preventing oxidative degradation [43]. Notably, the protective effects of ASX were more pronounced with pretreatment compared to co-treatment, suggesting that bolstering endogenous antioxidant defenses before toxicant exposure enhances mitochondrial resilience and reduces apoptotic susceptibility. This aligns with previous evidence showing that antioxidant priming improves cellular resistance to oxidative stress.

This study demonstrated that ASX modulated the expression of genes associated with oxidative stress, apoptosis, and inflammation signaling in CPF-exposed lung epithelial cells. Pretreatment was associated with a more pronounced upregulation of antioxidant and cytoprotective genes, including NRF2, HO-1, and BCL2, which may indicate a pre-conditioning effect enhancing the cellular defense capacity prior to toxicant exposure. In contrast, co-treatment was linked to a greater suppression of pro-apoptotic and inflammatory genes such as BAX, IL-6, and CASP3, suggesting a direct interference with CPF-induced molecular responses during exposure. Pro-inflammatory cytokines, including IL-1β, TNF-α, and IL-6, together with the transcription factor NFκB, are recognized as key regulators of the inflammatory signaling cascade. Upon exposure to toxic agents, the early release of TNF-α and IL-1β can initiate NFκB activation, which subsequently enhances the transcription of IL-6 and other inflammatory mediators, thereby establishing a self-amplifying regulatory cycle that sustains the inflammatory response [44]. CPF has been reported to induce lung injury accompanied by elevated IL-6, IL-1β, TNF-α, and NFκB levels [45,46]. Previous in vivo and in vitro studies have shown that ASX can inhibit NFκB nuclear translocation and reduce pro-inflammatory cytokine synthesis [47,48]. In our study, the gene expression profiles suggest that ASX may exert anti-inflammatory effects against CPF-induced responses in lung epithelial cells. CPF exposure also increased the transcription of pro-apoptotic genes (Caspase-3 and BAX) while reducing anti-apoptotic BCL2 levels, consistent with the activation of the mitochondrial-mediated apoptotic pathway. ASX treatment, particularly in the pretreatment group, was associated with partial reversal of these transcriptional alterations, which may reflect attenuation of apoptosis initiation. Moreover, the downregulation of CPF-induced VDAC1 and BAX expression, accompanied by restoration of BCL2, aligns with the potential preservation of mitochondrial membrane potential (MMP) and inhibition of apoptotic signaling. These transcriptional changes were paralleled by reduced LDH release, suggesting preservation of plasma membrane integrity and overall cellular homeostasis. CPF-induced DNA fragmentation, a characteristic feature of apoptosis, is often mediated through p53 activation [49,50,51]. In the present study, ASX pretreatment was associated with reduced DNA fragmentation and moderated p53 expression levels, which may contribute to the maintenance of genomic stability in CPF-exposed cells.

Intracellular Ca^2+^ homeostasis plays a critical role in maintaining mitochondrial function and overall cellular health. ICP-OES was chosen for its ability to sensitively quantify total elemental calcium under oxidative conditions. This technique measures total elemental Ca^2+^ rather than cytosolic free calcium and it serves as a reliable indicator of calcium overload and disturbed homeostasis under toxic stress conditions. Our results demonstrated that CPF exposure significantly elevated intracellular Ca^2+^ levels in A549 lung cells, indicating disruption of calcium homeostasis. The observed reduction in calcium accumulation following ASX treatment suggests its stabilizing effect on intracellular calcium regulation. Previous studies have shown that excessive intracellular Ca^2+^ induces mitochondrial dysfunction by promoting mPTP opening, resulting in membrane potential loss, impaired ATP production, ROS generation, and activation of apoptotic pathways [52]. ASX supplementation, especially pretreatment, markedly attenuated CPF-induced Ca^2+^ dysregulation, suggesting that ASX supports the preservation of calcium homeostasis and mitochondrial integrity. This protective effect is consistent with previous studies reporting ASX’s capacity to modulate Ca^2+^ signaling and inhibit Ca^2+^-mediated mitochondrial damage in oxidative stress-induced lung cells [53]. By stabilizing intracellular Ca^2+^ levels, ASX likely contributes to the prevention of mitochondrial permeability transition and subsequent apoptosis, thereby enhancing cell survival under CPF-induced stress.

A study conducted by Zárate et al. [54] demonstrated that low environmental doses of CPF increased VEGF-A and COX-2 expression and nitric oxide production in MCF-7 breast cancer cells, leading to enhanced vascular density and tubulogenesis in vivo. In the ovo CAM assay, CPF exposure resulted in evident vascular abnormalities, including focal hemorrhage, coagulation, and partial vessel lysis. These findings suggest that CPF may act as a vascular irritant, disrupting the physiological balance of angiogenesis. The vascular disruptions observed in the CAM assay, including hemorrhage, coagulation, and partial vessel degradation in the CPF group, appear consistent with oxidative stress, inflammation, and apoptosis detected in lung cells. These shared toxicological outcomes suggest that CPF-induced cellular dysfunction in the lung epithelium may translate into vascular injury in vivo. The restoration of vascular integrity upon ASX treatment further supports its protective effects observed at the molecular and cellular levels in the lung cell model. Moreover, pretreatment with ASX mitigated most of these CPF-induced vascular abnormalities, supporting its anti-inflammatory and antioxidant properties. The improved vascular pattern and reduced hemorrhagic lesions in the pretreatment group suggest that ASX can preserve vascular homeostasis and promote controlled angiogenic responses under toxic stress [55].

### Limitations and Future Perspectives

This study provides novel insights into the protective effects of astaxanthin (ASX) against CPF-induced oxidative injury in A549 lung epithelial cells, particularly through the combined evaluation of mitochondrial function, redox balance, and calcium homeostasis. However, certain limitations should be acknowledged. First, the experiments were conducted in a single human lung cell line, which may not accurately mimic or fully reproduce the complex in vivo microenvironment of respiratory tissues. Future studies should extend these findings to multiple cell types, including primary lung epithelial cells, and validate the protective role of ASX in animal models of respiratory CPF exposure. Second, while calcium measurements were conducted using ICP-OES to assess total intracellular calcium content, this method does not differentiate between cytosolic free Ca^2+^ and compartmentalized Ca^2+^ pools, which are critical for mitochondrial permeability and apoptosis regulation. Live-cell fluorescence imaging using probes such as Fura-2 AM or Fluo-4 could provide valuable complementary information on dynamic calcium signaling. Third, although our results suggest modulation of apoptosis- and antioxidant-related pathways, the lack of Western blot, ELISA, or confocal microscopy limits mechanistic confirmation at the protein and organelle levels. Future research should include such analyses to verify pathway activation and localize molecular events. In addition, advanced target discovery technologies, such as PROTAC-based probes and quantitative proteomics, could be employed to identify novel ASX-interacting proteins and delineate their precise molecular targets [56]. Finally, the bioavailability and pharmacokinetics of ASX in humans remain important considerations for translational relevance; therefore, pharmacological optimization and delivery strategies should be explored alongside in vivo toxicological assessments.

## 5. Conclusions

This study provided compelling evidence that ASX exerted potent protective effects against CPF-induced cytotoxicity in A549 lung cells and the CAM model. ASX, especially when applied as a pretreatment, effectively restored antioxidant defenses (SOD, GPx, TAC), attenuated oxidative damage markers (ROS, TOS, MDA, LDH), and mitigated mitochondrial impairment and calcium dysregulation. Additionally, ASX modulated critical apoptotic and inflammatory gene expressions, including upregulation of anti-apoptotic and antioxidant genes, alongside downregulation of pro-apoptotic and pro-inflammatory markers in CPF-induced lung cells. These data collectively reveal that ASX showed a multifaceted cytoprotective action by stabilizing mitochondrial redox homeostasis and regulating apoptosis and inflammation pathways.

Our findings provide the first comprehensive evidence of ASX’s ability to counteract CPF-induced lung epithelial injury, highlighting its potential as a therapeutic candidate for mitigating organophosphate pesticide-associated pulmonary toxicity. Given the well-documented toxicity of CPF in environmental and occupational settings, the use of ASX may offer a novel protective strategy. Furthermore, the integration of ASX into antioxidant-based therapies could contribute to the development of preventive or complementary treatments for chemically induced lung damage and associated inflammatory diseases. Importantly, the pronounced efficacy observed in the pretreatment regimen suggests that ASX administration could offer protective benefits in occupational or environmental settings involving anticipated CPF exposure, as well as support its use as a potential nutritional preventive strategy in high-risk populations. Further investigations using animal models and human systems are required to evaluate the long-term safety, bioavailability, efficacy, pharmacokinetics, and optimal dosing of these treatments to determine whether the findings can be effectively translated into clinical approaches for CPF-induced lung disorders.

## Figures and Tables

**Table 1:** Technical specifications of the commercial kits used in this study.

Assay/Parameter	Kit Name	Brand/Manufacturer	Catalogue No.	Method Principle	Units Reported
LDH release	LDH Cytotoxicity Assay Kit	Abcam, Cambridge, USA	ab102526	Colorimetric	µU/mL
TAC	Total Antioxidant Capacity Assay Kit	Rel Assay Diagnostics, Turkey	RL0017	Colorimetric	mmol Trolox eq./L
TOS	Total Oxidant Status Assay Kit	Rel Assay Diagnostics, Turkey	RL0024	Colorimetric	µmol H_2_O_2_ eq./L
ROS (IROS)	CellROX^®^ Green Reagent	Thermo Fisher Scientific, USA	C10444	Fluorescent probe oxidation by ROS	MFI
MDA	Lipid Peroxidation (MDA) Assay Kit	Abcam, Cambridge, USA	ab118970	Colorimetric	nmol/mg protein
SOD	Superoxide Dismutase Activity Assay Kit	Abcam, Cambridge, USA	ab65354	Colorimetric	U/mg protein
GPx	Glutathione Peroxidase Assay Kit	Abcam, Cambridge, USA	ab102530	Colorimetric	U/mg protein
Intracellular Ca^2+^	In-house method			ICP-OES (Thermo Scientific iCAP 7000 Series)	ng/10^6^ cells
DNA fragmentation	Diphenylamine (DPA) method			Colorimetric	% of total DNA
Mitochondrial Membrane Potential (MMP)	Safranin O quenching-mode assay/MITOISO1 mitochondrial isolation kit	Sigma-Aldrich, USA	MITOISO1	Fluorometric	MFI

## Data Availability

The original contributions presented in this study are included in the article/supplementary material. Further inquiries can be directed to the corresponding author(s).

## Supplementary Materials

The supporting information can be downloaded at https://www.mdpi.com/article/10.3390/cimb47080663/s1.

## Abbreviations

CPF	Chlorpyrifos
ASX	Astaxanthin
LDH	Lactate dehydrogenase
TAC	Total Antioxidant Capacity
TOS	Total Oxidative Stress
SOD	Superoxide dismutase
MDA	Malondialdehyde
GPx	Glutathione peroxidase
HET-CAM	Hen’s Egg Test Chorioallantoic Membrane
