# Supplementary material for: Astaxanthin Attenuates Chlorpyrifos-Induced Pulmonary Cytotoxicity by Modulating Mitochondrial Redox and Inflammatory Pathways
Source: Curr Issues Mol Biol. 2025 Aug 17;47(8):663. doi: 10.3390/cimb47080663 (PMC12384957; doi:10.3390/cimb47080663)
Supplement: Supplementary file 1 [file cimb-47-00663-s001.zip › cimb-3807013-supplementary.pdf]

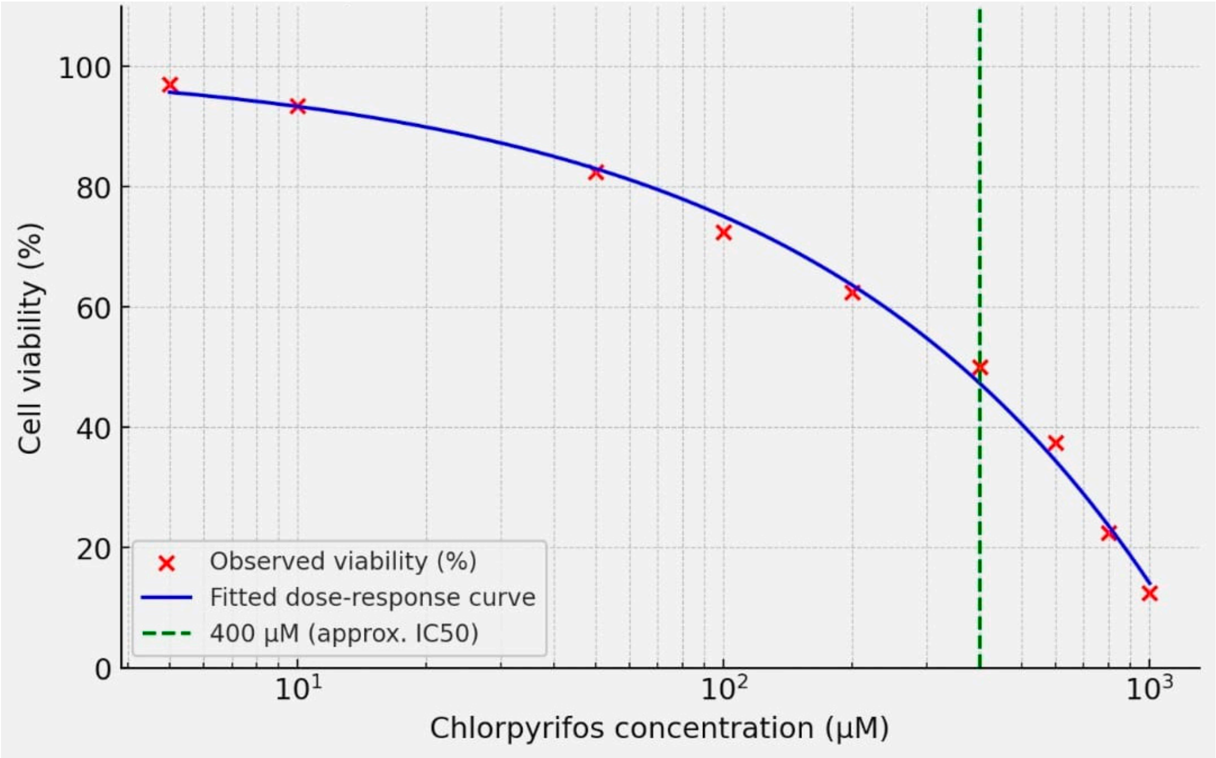

Figure S1. Dose–response curve for chlorpyrifos (CPF) on A549 cell viability. A549 cells were exposed to increasing concentrations of CPF (5–1000 µM) for 24 h, and cell viability was determined using the MTT assay. Red crosses represent the observed viability values (%), and the black curve indicates the fitted non-linear dose–response model. The vertical black dashed line marks the approximate IC_{50} value (400 µM), corresponding to the CPF concentration that reduced cell viability by ~50%.
